# C-reactive protein as a tool for monitoring response to treatment in dogs with acute hemorrhagic diarrhea syndrome

**DOI:** 10.3389/fvets.2022.1019700

**Published:** 2023-01-11

**Authors:** Florian Sänger, Stefan Unterer, Melanie Werner, René Dörfelt

**Affiliations:** ^1^Faculty of Veterinary Medicine, Clinic of Small Animal Medicine, Centre for Clinical Veterinary Medicine, Ludwig-Maximilians-University of Munich, Munich, Germany; ^2^Vetsuisse Faculty, Clinic for Small Animal Internal Medicine, University of Zurich, Zürich, Switzerland

**Keywords:** CRP, AHDS, CHDS index, APPLE score, sepsis, antibiotics

## Abstract

**Objectives:**

C-reactive protein (CRP) is an established marker for systemic inflammation in dogs that is especially elevated in dogs with sepsis. Some dogs with acute hemorrhagic diarrhea syndrome (AHDS) develop bacterial translocation and consequent sepsis during hospitalization. This study aimed to evaluate the course of CRP plasma concentrations during hospitalization and its correlation with clinical and other laboratory variables in dogs with AHDS.

**Methods:**

In this prospective, observational study, CRP was evaluated on days 0, 1, 2, and 3 in 27 client-owned dogs who presented with AHDS. Clinical examination data, blood pressure, acute patient physiologic and laboratory evaluation (APPLE) full and APPLE fast scores, and canine hemorrhagic diarrhea severity (CHDS) index were measured on the same days to evaluate the severity of the disease.

**Results:**

Twenty-five of the 27 dogs were discharged from hospital. Nineteen dogs received antimicrobial treatment due to sepsis or neutropenia. CRP values were mildly elevated on day 0 (median 27.3 mg/L; 1.0–125.8 mg/L) and markedly elevated on day 1 (median 88.9 mg/L; 1.4–192.7 mg/L). CRP concentrations decreased gradually over the following days. Moreover, CRP concentrations correlated moderately with albumin, leucocyte count, neutrophil count, and APPLE full and fast scores, but not with antimicrobial treatment.

**Conclusion and relevance:**

CRP concentrations were significantly elevated in patients with AHDS. In this study population, CRP did not help in detecting the requirement of antimicrobial treatment in dogs with AHDS. Nevertheless, as CRP can monitor the response to treatment, regular analysis can guide treatment.

## 1. Introduction

Acute hemorrhagic diarrhea syndrome (AHDS) is characterized by a sudden occurrence of bloody diarrhea and vomiting, with subsequent hemoconcentration due to severe dehydration. The etiology of AHDS is still unclear; however, it is suspected that a bacterial overgrowth of *Clostridium perfringens* and a release of its toxins (predominantly NetF-toxin) leads to the destruction of the intestinal mucosa and is responsible for the acute onset of clinical signs (1).

Blood in the feces reflects damage to the intestinal mucosa, which implies that the mucosal barrier is reduced. This can lead to an increased risk of bacterial translocation from the gut lumen into the systemic circulation and, potentially, sepsis (1). Patients with AHDS often present with signs of shock (reduced mental status, hypothermia, tachycardia, and tachypnea). However, prior to rehydration, it can be difficult to determine if these signs are caused by hypovolemic shock or systemic inflammation and sepsis, as these clinical signs are characteristic of both. Early recognition of sepsis is essential in order to establish an appropriate treatment plan.

Human consensus statements define sepsis as a systemic inflammation (systemic inflammatory response syndrome, SIRS) that is secondary to a suspected or confirmed infection (2, 3). Criteria for identification of SIRS are hyper- or hypothermia, tachycardia, tachypnea, and elevated or reduced leucocyte count. These criteria also apply to veterinary medicine (4). However, these parameters are not specific for sepsis and can also occur due to non-inflammatory processes, such as hypovolemic shock, excitement, or pain. Therefore, attempts to establish more specific parameters for identifying SIRS and sepsis have been made. One parameter is C-reactive protein (CRP), which is an acute-phase protein that can be severely elevated in patients with SIRS or sepsis as compared to healthy patients. However, some studies have shown that CRP is not specific for the diagnosis of sepsis; however, its degree of elevation and course is important for interpretation of the disease (5).

Another assessment tool for critically ill patients is the acute patient physiologic and laboratory evaluation (APPLE) score. The APPLE full score consists of 10 criteria (creatinine, white blood cell count, albumin, total bilirubin, age, respiratory rate, lactate, SpO_2_, fluid score, and mentation score) and the APPLE fast score consists of 5 criteria (glucose, albumin, lactate, platelet count, mentation score). The scores can be used to estimate the probability of survival that is independent of the disease (6).

The canine hemorrhagic diarrhea severity (CHDS) index has been established especially for AHDS patients, and has been implemented in several studies (1, 7, 8). It consists of six criteria, and aims to determine the severity of illness in dogs with AHDS (7). However, the knowledge about the use of CRP in dogs with AHDS is limited. One study compared CRP values in dogs with AHDS among patients who received antimicrobial treatment and patients who did not. Dogs that received antimicrobial treatment had higher CRP values compared to dogs that did not receive any antimicrobial treatment. However, the limitations of this study were its retrospective study design, and that CRP might have been used in deciding when to implement antimicrobial treatment (9).

The primary aim of this study was to evaluate whether CRP concentrations increase in patients with AHDS and whether they correlate with the clinical course, duration of hospitalization, and clinical and laboratory variables, which are considered markers of disease severity and sepsis in dogs with AHDS. The secondary aim was to evaluate the potential of CRP in the diagnosing requirement of antimicrobial treatment due to SIRS or neutropenia in AHDS upon admission. We hypothesized that CRP concentrations are elevated in patients with AHDS, which may help in differentiating patients who need antimicrobial treatment from those with an uncomplicated disease course and no need of antimicrobial treatment at presentation.

## 2. Materials and methods

### 2.1. Ethical approval

This study was approved by the ethical committee of the Center of Clinical Veterinary Medicine of the Ludwig-Maximilians-University Munich (approval no. 186-23-09-2019). Dog owners signed an informed consent form before participating in the study.

### 2.2. Inclusion criteria

The study population consisted of client-owned dogs that presented with AHDS. Dogs were included if the duration of acute hemorrhagic diarrhea was < 3 days, hemoconcentration occurred with a hematocrit ≥50 %, and a CHDS index ≥6 was present. Hospitalization for at least 24 h was required.

### 2.3. Exclusion criteria

Underlying diseases known to cause hemorrhagic diarrhea (e.g., parvovirosis, giardiosis, other parasitosis, ingestion of non-steroidal anti-inflammatory drugs) except involvement of *C. perfringens* were ruled out with fecal examination, parvovirus snap test, and patient history. Patients with parvovirus, obstructive bowel disease, or any disease requiring surgical intervention (e.g., foreign body ileus, intussusception) were excluded from this study. Evidence of potentially enteropathogenic bacteria was not an exclusion criterion.

### 2.4. Study design

During this prospective observative clinical study, dogs were evaluated when presented to the hospital (day 0) and clinical examination, pulse oximetry, non-invasive blood pressure, hematology, blood gas analysis, and point-of-care sonography using the A-FAST and T-FAST protocols (10) were performed. Further, the biochemistry profile and CRP concentrations were obtained. Thereafter, the CHDS index was calculated as described in previous publications (1). APPLE fast and full scores were calculated as described before (6). All examinations and scores were performed and calculated daily during the first 3 days of hospitalization (days 1, 2, and 3).

Some parameters could not be determined in all patients at all timepoints due to technical issues of the laboratory analyzer. Problems could not be solved out of hours. As the number of dogs decreased gradually from day 0 to day 3 due to clinical improvement and hospital discharge, only 25/36 patients remained on day 3. One dog died on day 1 and another dog was euthanized after clinical deterioration at the owner's request. Two dogs were discharged before day 2 and three dogs were discharged before day 3.

Hematology was analyzed with a Sysmex XT-2000i (Sysmex Deutschland GmbH, Germany), biochemistry profile was analyzed with a Cobas Integra 400 Plus analyzer (Roche Diagnostics, Basel, Switzerland), and CRP concentration was measured with immunoturbidimetry (Cobas Integra 400 Plus analyzer, Roche Diagnostics) from serum samples of patients as previously described in dogs (11). Neutrophils were manually counted. CRP results were blinded to the clinician and the observers of this study so that the treatment decisions, especially antimicrobial treatment, were not influenced by CRP values.

Additional examinations to rule out other individual underlying diseases included fecal flotation, point-of-care *Giardia* antigen enzyme-linked immunosorbent assay (ELISA) (Snap Giardia; IDEXX Laboratories, Westbrook, ME), basal cortisol, point-of-care canine pancreatic-specific lipase test (cPL snap test; IDEXX Laboratories), parvovirus snap test (Snap Parvo; IDEXX Laboratories) and parvovirus PCR test of feces. A parvovirus test was only performed if patients had appropriate anamnestic and clinical signs, or neutropenia. These additional examinations were not performed in all patients.

All patients received fluid therapy with lactated Ringer's solution (Ringer-Lactate; B.Braun Vet Care, Tuttlingen, Germany) as a 30 mL/kg shock bolus intravenously over 10 min, which was repeated once or twice if necessary. Thereafter, the infusion was continued to compensate for dehydration, ongoing fluid losses, and maintenance fluid requirements. Buprenorphine (10 μg/kg IV q 6–8 h; Bupresol, CP-Pharma, Burgdorf, Germany) or metamizole (40 mg/kg IV q 8 h; Novaminsulfon, Dechra Pharmaceuticals, Northwich, UK) were administered for analgesia, depending on the patient's requirements. All patients were anorectic and vomiting at the time of presentation and were treated with antiemetics. At the least, maropitant (1 mg/kg IV q 24 h; Prevomax, Dechra Pharmaceuticals) was administered in every patient. Metoclopramide at a constant rate infusion of 80 μg/kg/h IV (Emeprid; Ceva, Libourne, France) and ondansetron (0.2 mg/kg IV q 8 h; Ondansetron-hameln, Hameln Pharma Plus, Hameln, Germany) were administered if patients were vomiting despite maropitant treatment. In addition, esomeprazole (1 mg/kg IV q 12 h; Nexium; Grünenthal, Aachen, Germany) and sucralfate (30 mg/kg PO q 8 h; Sucrabest, Combustin, Hailtingen, Germany) were used as gastrointestinal protectants according to individual assessment. If dogs were considered to develop sepsis, amoxicillin clavulanic acid (20 mg/kg IV q 8 h; Amoxiclav Hexal 500/100 mg, Hexal, Holzkirchen, Germany) was given as first-line antimicrobial treatment. In severely affected patients, marbofloxacin (4 mg/kg IV q 24 h; Marbocyl, Vetoquinol, Lure, France) was started as an additional antimicrobial therapy after amoxicillin clavulanic acid and reduced to 2 mg/kg IV q 24 h on the following days.

Criteria for developing sepsis were a minimum of two of the following: persistent tachycardia >140 bpm, hyperthermia >39.5°C or hypothermia < 37.5°C after treating shock, neutrophilia >25 × 10^9^/L, neutropenia < 3 × 10^9^/L, elevated band cells >2 × 10^9^/L, leucopenia < 5 × 10^9^/L, hypoglycemia < 3.9 mmol/L, hyperbilirubinemia >5.3 μmol/L, or other evidence of organ dysfunction. Criteria were adapted from previous publications reporting SIRS criteria (9, 12). Antimicrobial therapy was initiated if a patient was classified as septic or if there was severe neutropenia (< 2 × 10^9^/L). Before initiating antimicrobial treatment, a single sterile blood sample of 10 mL from the jugular vein was collected for bacteriological culture in a blood culture medium (Oxoid signal blood culture system medium; Thermo Fisher Scientific, Waltham, MA) and analyzed for bacterial growth and antimicrobial susceptibility. Criteria for hospital discharge were normal clinical parameters and the absence of blood in the feces.

### 2.5. Statistical analysis

Statistical analysis was performed using commercially available software (Prism 5 for Windows; GraphPad Software, San Diego, CA). Data were evaluated for normality with the D'Agostino & Pearson normality test. Parametric data were reported as mean ± standard deviation. Non-parametric data were reported as median and range.

CRP concentrations and other laboratory variables obtained on different days of hospitalization were compared using a Friedman test, and Dunn's multiple comparison test was used for *post hoc* analysis. Δ-values were calculated as the difference between days 0 and 1.

Correlation of CRP concentrations with other clinical and laboratory variables, difference in CRP concentrations (ΔCRP) with other variables, and changes in other variables were analyzed using Spearman correlation for non-parametric variables. The CRP values of dogs that received antimicrobial treatment were compared with those that did not receive antimicrobial treatment using the Mann–Whitney U test. Changes in the variables during hospitalization were analyzed using the Kruskal–Wallis test with Dunn's multiple comparison test. The parameters of dogs receiving antimicrobial treatment and dogs not receiving antimicrobial treatment were presented as mean ± standard deviation and were analyzed using a *t*-test if the data was normally distributed. If the data was not normally distributed, they were presented as median (min–max), and were analyzed using the Mann–Whitney U test. A *P* ≤ 0.05 was considered significant.

## 3. Results

The study population consisted of 36 dogs with AHDS. Nine dogs were excluded from the study because of missing parameters in the laboratory analysis. The remaining 27 dogs included six intact males, six neutered males, 11 intact females, and four spayed females. The five most common breeds were mixed breed (10/27), Zwergpinscher (2/27), Labrador Retriever (2/27), Cocker Spaniel (2/27), and Jack Russell Terrier (2/27). All other breeds were represented with one dog each. The mean age was 6.5 ± 3.4 years. The median weight was 8.5 kg (3.4–60.0 kg).

Twenty-five of the 27 dogs were discharged from the hospital. One dog died during hospitalization and one dog was euthanized after clinical deterioration at the owner's request. The two dogs died within the first 48 h of the study. The mean duration of hospitalization was 4.3 ± 1.5 days for the surviving dogs. Nineteen dogs were categorized as septic and received antimicrobial treatment, and bacteriological blood culture was performed in 17 of these dogs. Two blood cultures were positive for *C. perfringens*.

Fecal flotation and point-of-care *Giardia* antigen ELISA were performed in 14/27 dogs. Basal cortisol was assessed in 12/27 dogs. Point-of-care canine pancreatic-specific lipase tests were performed in 3/27 dogs. Parvovirus snap tests and parvovirus PCR tests of feces were performed in 2/27 dogs.

At presentation, 21 of the 27 dogs had elevated CRP values >10 mg/L compared to the reference interval. The median CRP was mildly elevated (27.3 mg/L; 1.0–125.8 mg/L) at presentation, increased on day 1 (88.9 mg/L; 1.4–192.7 mg/L), and decreased at day 3 (30.2 mg/L; 6.2–162.0 mg/L). Upon analyzing only dogs with four CRP values with a Friedman test, CRP was significantly higher at day 1 compared to days 0 or 3 (*p* < 0.001) (Table 1). Serial monitoring of CRP and other clinicopathological variables from hospital admission to day 3 were available for 17 of 27 dogs (Table 1).

**Table 1:** Laboratory variables, and clinical scores of 17 dogs with acute hemorrhagic diarrhea syndrome (ADHS) evaluated upon admission and during the course of the disease.

	**Reference interval**	***N***	**Day 0**	**Day 1**	**Day 2**	**Day 3**	***p***
CRP (mg/L)	< 10.0	17	27.3 (1.0–125.8)	88.9 (1.4–192.7)^*^	59.8 (9.0–114.6)	30.2 (6.2–162.0)^#^	< 0.001
Albumin (g/L)	31.3–43.0	17	36.8 (29.8–46.6)	25.9 (17.8–41.6)^*^	25.6 (19.4–43.4) ^*^	28.3 (19.4–44.3)^*^	< 0.001
Glucose (mmol/L)	4.1–7.1	17	5.3 (2.4–9.8)	5.4 (4.4–7.9)	5.7 (4.4–7.6)	5.8 (4.8–8.1)	0.708
Total bilirubin (μmol/L)	< 5.26	17	0.8 (0.0–4.2)	1.0 (0.0–9.2)	1.3 (0.2–46.2)	1.2 (0.1–103.9)	0.628
Leucocyte count ( × 10^9^/L)	5.0–16.0	17	9.2 (3.3–26.0)	6.2 (1.7–27.1)	9.8 (3.4–31.4) ^#^	11.1 (5.2–43.4) ^#^	0.016
Neutrophil count ( × 10^9^/L)	3.0–9.0	17	7.1 (1.9–23.7)	3.8 (0.7–21.0)^*^	6.9 (2.0–26.2)	6.9 (2.1–37.4)^#^	0.003
Hematocrit value (%)	35.0–58.0	17	56.9 (50.7–70.0)	46.1 (39.7–57.5)^*^	45.7 (37.1–52.0)^*^	42.5 (34.1–50.9)^*^	< 0.001
CHDS index		17	16.0 (12.0–18.0)	13.0 (5.0–16.0)	7.0 (2.0–16.0)^*^	4.0 (1.0–15.0)^*, #^	< 0.001
APPLE full score		17	15.0 (4.0–39.0)	20.0 (7.0–31.0)	18.0 (6.0–26.0)	17.0 (6.0–22.0)	0.163
APPLE fast score		17	15.0 (10.0–36.0)	23.0 (14.0–26.0)	21.0 (16.0–26.0) ^*^	17.0 (12.0–24.0)	0.007

Data are reported as median and range and analyzed using the Friedman test with Dunn's multiple comparison test.

N, number of dogs; CRP, C-reactive protein; CHDS, canine hemorrhagic diarrhea severity; APPLE, acute patient physiologic and laboratory evaluation.

^*^Significantly different from day 0.

^#^Significantly different from day 1.

The initial CRP concentration correlated negatively with albumin concentrations, leucocyte count, and neutrophil count, and correlated positively with the APPLE full and fast scores on day 0. Correlations for all variables were moderate. No significant correlations were observed with glucose, total bilirubin, hematocrit, CHDS index, or duration of hospitalization (Table 2). The duration of hospitalization was calculated without the two dogs that did not survive.

**Table 2:** Spearman correlation of C-reactive protein (CRP) with laboratory variables and clinical scores of 27 dogs with acute hemorrhagic diarrhea syndrome (AHDS) at presentation.

**Correlation of CRP with**	***N***	**Correlation coefficient**	***P* value**
Albumin	27	−0.500	0.008^*^
Glucose	27	0.134	0.489
Total bilirubin	27	0.190	0.343
Leucocyte count	27	−0.469	0.013^*^
Neutrophil count	27	−0.554	0.003^*^
Hematocrit value	27	0.006	0.976
CHDS index	27	−0.086	0.668
APPLE full score	27	0.602	0.001^*^
APPLE fast score	27	0.479	0.012^*^
Duration of hospitalization	27	0.017	0.936

N, number of dogs; CRP, C-reactive protein; CHDS, canine hemorrhagic diarrhea severity; APPLE, acute patient physiologic and laboratory evaluation.

Significant correlations are marked with ^*^.

From presentation to day 1, ΔCRP correlated negatively with Δleucocyte count and Δneutrophil count, and positively with ΔAPPLE full score and ΔAPPLE fast score. However, no significant correlation was observed with Δalbumin, Δglucose, Δtotal bilirubin, Δhematocrit, or ΔCHDS index (Table 3).

**Table 3:** Correlation of Δ C-reactive protein (day 0–day 1) with different variables.

**Correlation of Δ CRP (day 0–day 1) with**	***N***	**Correlation coefficient**	***P* value**
**Δ** Albumin (day 0–day 1)	27	−0.316	0.102
**Δ** Glucose (day 0–day 1)	27	0.313	0.105
**Δ** Total bilirubin (day 0—day 1)	27	0.269	0.167
**Δ** Leucocyte count (day 0—day 1)	27	−0.485	0.009^*^
**Δ** Neutrophil count (day 0—day 1)	27	−0.519	0.005^*^
**Δ** Hematocrit value (day 0—day 1)	27	−0.246	0.207
**Δ** CHDS index (day 0—day 1)	27	0.224	0.253
**Δ** APPLE full score (day 0—day 1)	27	0.428	0.023^*^
**Δ** APPLE fast score (day 0—day 1)	27	0.597	0.001^*^

N, number of dogs; CRP, C-reactive protein; CHDS, canine hemorrhagic diarrhea severity; APPLE, acute patient physiologic and laboratory evaluation.

Significant correlations are marked with ^*^. Correlation was analyzed using Spearman correlation.

In dogs receiving antimicrobial treatment, the duration of hospitalization was significantly longer than in dogs not receiving antimicrobial treatment (p = 0.014) (Table 4). Other variables (ΔCRP, glucose, total bilirubin, albumin, leucocyte count, neutrophil count, CHDS index, APPLE full score, APPLE fast score) were not significantly different between these two groups.

**Table 4:** Comparison of different parameters at presentation between patients with and without antimicrobial treatment.

**Parameter**	***N***	**No antimicrobial treatment**	***N***	**Antimicrobial treatment**	***P*-value**
CRP (mg/L)	8	25.0 ± 26.3	19	70.8 ± 63.5	0.062
Δ CRP (mg/L)	8	28.3 ± 28.7	19	22.1 ± 32.1	0.837
Heart rate	8	157 ± 43	19	148 ± 45	0.661
Respiratory rate	8	28 (20–32)	19	28 (20–60)	0.167
Temperature (°C)	8	38.5 ± 0.5	19	38.1 ± 1.1	0.430
Glucose (mmol/L)	8	5.9 ± 1.1	19	5.4 ± 1.7	0.454
Total bilirubin (μmol/L)	8	1.3 (0.1–2.2)	19	0.9 (1.0–4.5)	0.831
Albumin (g/L)	8	37.7 ± 2.9	19	35.8 ± 6.2	0.441
Leukocyte count ( × 10^9^/L)	8	10.9 ± 2.7	19	11.1 ± 6.5	0.950
Neutrophil count ( × 10^9^/L)	8	9.1 ± 2.6	19	8.7 ± 6.3	0.847
Hematocrit value (L/L)	8	0.574 ± 0.035	19	0.595 ± 0.063	0.397
CHDS index	8	14.9 ± 2.4	19	15.8 ± 1.8	0.279
APPLE full score	8	15 (9–21)	19	18 (4–51)	0.093
APPLE fast score	8	15.1 ± 3.2	19	18.3 ± 7.2	0.243
Duration of hospitalization	8	3.3 ± 0.7	19	4.8 ± 1.6	0.014 ^*^

N, number of dogs; CRP, C-reactive protein; Δ CRP, difference in CRP values between day 0 and day 1; CHDS, canine hemorrhagic diarrhea severity; APPLE, acute patient physiologic and laboratory evaluation. Normally distributed data are presented as mean ± standard deviation and were analyzed using a t-test; non-normally distributed data are presented as median (min–max), and were analyzed using the Mann–Whitney U test. Significant values are marked with ^*^.

**Figure 1:**
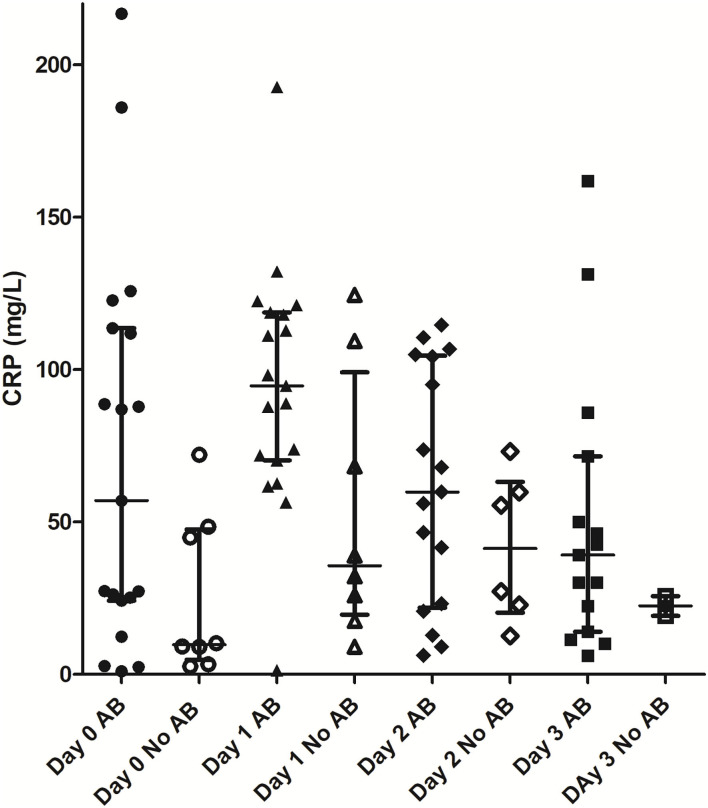
Comparison of CRP values between patients with and without antimicrobial treatment. AB, antimicrobial treatment; CRP, C-reactive protein.

Mean CRP concentrations in dogs receiving antimicrobial treatment (*n* = 19/27) tended to be higher compared to dogs without antimicrobial treatment (*n* = 8/27), however a statistically significant difference was not found (*p* = 0.062) (Table 4, Figure 1).

## 4. Discussion

The present study evaluated CRP concentrations in dogs with AHDS at presentation and during hospitalization. CRP concentrations at presentation did not differ between dogs requiring antimicrobial treatment based on the criteria elected in this study.

CRP values of the dogs at presentation were mildly elevated compared to the reference interval. As CRP concentrations peak within 24 h after the insult, they may not be severely elevated a few hours after the acute and sudden onset of AHDS. In dogs undergoing major surgery, an increase in CRP concentrations could be first detected 4 h after surgery (13). In another study, maximum CRP concentrations were attained 24 h after surgery (14).

In our study, CRP concentrations were markedly elevated with a median of 88.9 mg/L (1.4–192.7 mg/L) on day 1. This is in agreement with a retrospective study evaluating disease severity and outcome in dogs with AHDS (9). Severely elevated CRP concentrations have been detected in many systemic disorders, such as pyometra, acute pancreatitis, polyarthritis, immune-mediated diseases like immune-mediated hemolytic anemia, and neoplastic diseases like hemangiosarcoma. The median CRP values detected in these dogs ranged between 65 and >200 mg/L (15). Another study that evaluated different respiratory diseases in dogs detected the highest CRP values in patients with bacterial pneumonia, with a median of 121 mg/L (16). The highest CRP values seem to occur in dogs with severe bacterial infections, such as pyometra, with a median CRP value of 200 mg/L (15). In the present study, the median CRP concentration of 88.9 mg/L (1.4–192.7 mg/L) was not as high as in other diseases with bacterial infection. The etiology of AHDS is not fully clarified, but a bacterial overgrowth of *C. perfringens* and a subsequent production of *C. perfringens* toxins is suspected (17). Acute and severe hemorrhagic diarrhea is a consequence of necrosis of the intestinal mucosa (1, 18). Lesions are caused by bacterial toxins. Thus, AHDS does not represent a purulent bacterial infection, such as pyometra or pneumonia. *C. perfringens* is an enterotoxic pathogen that induces epithelial cell injury—it is not a mucosal invasive species. Thus, AHDS should not be viewed as a classical bacterial infection, since a transient overgrowth of clostridial strains is responsible for this syndrome (19). Without complications, AHDS presents as a self-limiting disorder. Many dogs rapidly improve within a few days with only fluid and symptomatic therapy, and antibiotics do not shorten the duration of disease in uncomplicated cases. This might be a reason for the overall lower CRP values in dogs with AHDS.

The mean age of the dogs in the present study was 6.5 ± 3.4 years. The median weight was 8.5 kg (3.4–60.0 kg). This is similar to previous studies as most dogs with AHDS are middle-aged, small-breed dogs, but every breed and sex can be affected (19).

Twenty-five of the 27 dogs (92.6%) in the present study survived and were discharged from the hospital. The mean duration of hospitalization was 4.3 ± 1.5 days. The mortality rate in AHDS is typically lower than 10% if patients are hospitalized and treated adequately (19). Clinical recovery occurs within 24–72 h in most patients (19). The duration of hospitalization in the present study was longer, most likely because more severely affected patients were included.

In the present study, 2 out of 17 bacteriological blood cultures were positive for *C. perfringens*. As mentioned before, clinical signs in AHDS are caused by bacterial toxins and not by *C. perfringens* itself (18, 19). One reason for the low number of positive cultures might be a high clearance of the bacteria from the circulation, but delayed clinical signs, after damage to the intestinal mucosa and necrosis due to the toxins.

The mean albumin concentration decreased from presentation to day 1 and stayed at this level for the following days. Patients with hemorrhagic diarrhea lose albumin with their feces. As another aspect, patients with AHDS are often hemoconcentrated and require large amounts of fluids. The application of high volumes of fluid leads to dilution of albumin. Severe hypoalbuminemia is an indication of a more severe disease process (19). Glucose and total bilirubin concentrations were not significantly different between timepoints. Both variables were used as sepsis markers, but changes could only be found in a few patients. This indicates that these variables could be used as additional markers for organ failure and sepsis but not as single criteria. Hematocrit value was increased at presentation and returned to the reference range on day 1 in all dogs. Hemoconcentration is a typical finding in patients with AHDS due to fluid loss through vomitus and diarrhea. With adequate volume resuscitation, hematocrit value normalizes quickly (19).

CRP did not correlate significantly with the CHDS index or duration of hospitalization. This might indicate that CRP is not an appropriate marker for disease severity or prognosis in AHDS. In contrast, a retrospective study of dogs with AHDS observed a significant correlation between CRP and CHDS index, but not between CRP and duration of hospitalization (9). These results might be biased due to the retrospective nature of the study. CRP could have been used as a decision criterion to start antimicrobial treatment and could have influenced the clinical disease course. In the present study, CRP measurements were blinded to the attending clinicians and therefore could not be used for therapeutical decisions. As the dogs in the present study had a comparatively high survival rate, an assessment of CRP as a predictive marker for survival was not possible.

The classification of cases as requiring antimicrobial treatment due to sepsis or not requiring antimicrobial treatment did not influence the CRP values based on the defined criteria of this study. As CRP is a non-specific marker of inflammation, other systemic inflammatory processes such as SIRS or neoplastic diseases can also cause an elevation in CRP similar to that in bacterial infections (5, 20). Most dogs with AHDS have severe necrosis of the intestinal mucosa, which leads to an inflammatory reaction with infiltration of primarily neutrophils, regardless of the septic processes. Therefore, CRP might be elevated in most AHDS patients due to widespread local inflammation of the entire intestinal tract, even without bacterial translocation or sepsis. This may explain the inability of CRP to differentiate between septic and non-septic dogs with AHDS. In addition, only severe cases hospitalized for at least 24 h were included, which might also bias the results and may explain the high CRP concentrations in both groups. The effect of additional treatment on CRP concentrations was not evaluated in our study. Demonstrating a difference in CRP concentrations among dogs with or without bacterial translocation was not possible, as only 2 of the 17 dogs had a positive blood culture.

Dogs receiving antimicrobial treatment had a significantly longer duration of hospitalization compared to dogs not receiving antimicrobial treatment despite not a lack of significant differences in CRP concentrations. The duration of hospitalization seems to reflect the severity of illness in these patients, as dogs with continuing hemorrhagic diarrhea and anorexia were hospitalized longer. As the clinicians were blinded to the CRP measurements and antimicrobial treatment was initiated due to the sepsis criteria of this study or due to neutropenia, this might support the assumption that CRP is not a good prognostic marker in patients with AHDS.

Nineteen of 27 dogs were categorized as septic and received antimicrobial treatment. This is in contrast to previous reports, where most patients recovered quickly within 24–72 h without antimicrobial treatment (19). The reason for the high percentage of septic patients in this study might be the inclusion criteria for the study. Only patients that were hospitalized for at least 24 h were included in this study. Mildly diseased dogs with AHDS do not need hospital treatment. Therefore, only more severe diseased patients were included in this study.

The present study has several limitations. There is no established definition for sepsis in veterinary patients, and the attending clinician's estimation had a major influence on the classification. Most veterinary studies use SIRS criteria and the suspicion of a bacterial infection to define sepsis. SIRS criteria include hypo- or hyperthermia, tachycardia, tachypnea, leukopenia, or leukocytosis and an elevated number of banded white blood cells (4, 21). However, these variables seem to not be useful in dogs with AHDS because most of them are hypovolemic at presentation, which may interfere with SIRS criteria regardless of the presence of sepsis. Depending on the patient's clinical course, ongoing fluid losses due to diarrhea and vomiting may contribute to hypovolemia. Parameters for defining sepsis in this study were SIRS criteria after shock therapy and less volume-dependent parameters like neutrophil count, glucose, or total bilirubin. However, some of these variables can change in AHDS without the presence of sepsis. For example, destruction of the mucosa over the whole length of the intestinal tract could lead to a significant consumption (mild to moderate neutropenia) with consecutive increased production (mild to moderate left-shift) of neutrophils. Therefore, the classification of patients as septic or non-septic might be wrong in some cases. This can lead to a wrong interpretation of the comparison of CRP results between septic and non-septic patients. The decision regarding administration of antimicrobial treatment depended on the clinician's estimation of the patient being septic or not or the presence of severe neutropenia. Due to the difficulty in defining sepsis, it is possible that some dogs received antimicrobial treatment even though they were not septic, and some dogs with bacterial translocation where antibiotics were indicated did not receive antimicrobial treatment. There was no clear definition for the use of marbofloxacin as a second antibiotic. Amoxicillin clavulanic acid was administered in all patients classified as septic intravenously as a first line antimicrobial treatment. If the patient still showed sepsis criteria 12 h after initial antimicrobial treatment, marbofloxacin was started additionally. Further, the diagnostic workup was not entirely uniform in all patients. All dogs received a standardized workup with clinical examination, pulse oximetry, non-invasive blood pressure, automated blood cell count, blood gas analysis, clinical chemistry, CRP, A-FAST, and T-FAST. However, supplementary examinations, such as fecal flotation, *Giardia* snap test, serum cortisol analysis, canine pancreatic-specific lipase snap test, parvovirus snap test, and parvovirus PCR test were only performed with clinical suspicion. Therefore, underlying disease processes other than AHDS might have been overlooked in some patients. The number of patients in this study is small. Due to problems with the laboratory analyzer, some patients had to be excluded from the study. Therefore, the number of patients might have been too small to detect a difference in CRP concentrations between dogs receiving antimicrobial treatment and dogs not receiving antimicrobial treatment.

In conclusion, CRP values were significantly elevated over the reference interval in patients with AHDS at presentation, increased after 1 day, and decreased during hospitalization in our study. Based on the sepsis criteria chosen in this study, there was no difference in CRP concentrations between septic and non-septic patients. In our study, CRP concentration was not useful as a prognostic variable for predicting the clinical course and outcome. Nevertheless, as CRP can monitor the response to treatment, regular analysis can guide treatment.

## Data availability statement

The original contributions presented in the study are included in the article/Supplementary material, further inquiries can be directed to the corresponding author.

## Ethics statement

The animal study was reviewed and approved by Ethical Committee of the Center of Clinical Veterinary Medicine of the Ludwig-Maximilians-University Munich. Written informed consent was obtained from the owners for the participation of their animals in this study.

## Author contributions

FS contributed to study design, sample collection, study analysis, and wrote the manuscript. RD contributed to study design, statistical analysis, and revision of the manuscript. SU contributed to study design and revision of the manuscript. MW contributed to revision of the manuscript. All authors contributed to the article and approved the submitted version.
