# Supplementary material for: C-reactive protein as a tool for monitoring response to treatment in dogs with acute hemorrhagic diarrhea syndrome
Source: Front Vet Sci. 2023 Jan 11;9:1019700. doi: 10.3389/fvets.2022.1019700 (PMC9874157; doi:10.3389/fvets.2022.1019700)
Supplement: Supplementary file 1 [file Table_1.DOCX]

Supplementary Material

# Supplementary Tables

**Supplementary Table 1.** Parameters of 27 dogs with acute hemorrhagic diarrhea syndrome (ADHS) at initial presentation and as basis for categorization as septic or non-septic.

| **Patient number** | **Breed** | **Heart rate (beats/min)** | **Respiratory rate (breaths/min)** | **Temperature (°C)** | **Neutrophil count (****x 10^9^/L)** | **Total bilirubin (µmol/L)** | **Glucose (mmol/L)** | **Antibiotics** | **Sepsis** | **Reason for sepsis / organ dysfunction** |
| --- | --- | --- | --- | --- | --- | --- | --- | --- | --- | --- |
| 1 | German Shepherd | 120 | 36 | 38.5 | 3.4 | 4.5 | 5.1 | Yes | Yes | Septic on day 1; hyperbilirubinemia, prolonged coagulation times |
| 2 | Bichon Frise | 200 | 28 | 38.5 | 16.8 | 0.1 | 5.2 | Yes | Yes | Septic on day 1; neutropenia, hyperbilirubinemia |
| 3 | Puggle | 160 | 32 | 38.0 | 23.7 | 1.3 | 2.4 | Yes | Yes | Septic on day 0; hypoglycemia, elevated bands |
| 4 | Cavalier King Charles Spaniel | 140 | 32 | 37.7 | 3.4 | 0.6 | 6.0 | Yes | Yes | Septic on day 1; hypothermia, neutropenia, thrombocytopenia |
| 5 | Miniature pinscher | 180 | 20 | 37.6 | 7.1 | 0 | 4.1 | Yes | Yes | Septic on day 1; neutropenia |
| 6 | Pinscher | 144 | 36 | 38.8 | 5.9 | 0.6 | 4.9 | Yes | Yes | Septic on day 0; tachycardia, elevated bands |
| 7 | Crossbreed | 200 | 28 | 37.5 | 8.3 | 0.2 | 8.2 | Yes | Yes | Septic on day 1; neutropenia |
| 8 | Crossbreed | 160 | 24 | 38.1 | 12.1 | 0.8 | 3.9 | Yes | Yes | Septic on day 1;  hypothermia, elevated bands |
| 9 | Crossbreed | 200 | 36 | 36.3 | 15.8 | 0.2 | 5.7 | Yes | Yes | Septic on day 1; neutropenia,  hyperbilirubinemia, hypoglycemia |
| 10 | Jack Russell Terrier | 68 | 24 | 37.4 | 6.3 | 0.8 | 6.6 | Yes | Yes | Septic on day 1; hypothermia, neutropenia,  hyperbilirubinemia |
| 11 | Malinois | 72 | 32 | 39.0 | 18.7 | 4.2 | 5.3 | Yes | Yes | Septic on day 2; neutrophilia, elevated bands |
| 12 | Labrador Retriever | 152 | 46 | 38.7 | 9.4 | 2.0 | 4.1 | Yes | Yes | Septic on day 1; leucopenia |
| 13 | Small Münsterländer | 200 | 60 | 41.0 | 1.9 | 2.4 | 9.8 | Yes | Yes | Septic on day 0; neutropenia,  hyperbilirubinemia |
| 14 | Cocker Spaniel | 140 | 28 | 38.0 | 2.9 | 1.6 | 6.2 | Yes | Yes | Septic on day 0; neutropenia, elevated bands |
| 15 | Jack Russell Terrier | 124 | 32 | 39.5 | 8.5 | 1.4 | 6.6 | No | No |  |
| 16 | Crossbreed | 152 | 24 | 38.7 | 5.7 | 1.3 | 3.9 | No | No |  |
| 17 | Labrador Retriever | 100 | 24 | 38.8 | 7.7 | 1.3 | 5.7 | Yes | Yes | Septic on day 1; hypothermia, neutropenia  hyperbilirubinemia |
| 18 | Irish Wolfhound | 120 | 20 | 38.3 | 2.7 | 2.8 | 5.3 | Yes | Yes | Septic on day 0; neutropenia |
| 19 | Miniature pinscher | 240 | 28 | 38.6 | 12.9 | 2.2 | 5.1 | No | No |  |
| 20 | Crossbreed | 160 | 28 | 35.7 | 2.5 | 1.6 | 2.8 | Yes | Yes | Septic on day 0; neutropenia,  hypoglycemia |
| 21 | Crossbreed | 212 | 24 | 37.9 | 4.3 | 0.8 | 4.9 | Yes | Yes | Septic on day 1; hypothermia |
| 22 | Crossbreed | 132 | 20 | 37.8 | 7.0 | 0.3 | 6.4 | No | No |  |
| 23 | Cocker Spaniel | 120 | 28 | 37.9 | 11.2 | 1.3 | 5.3 | No | No |  |
| 24 | Crossbreed | 88 | 32 | 38.3 | 11.9 | 0.9 | 6.6 | Yes | Yes | Septic on day 2; hypothermia, elevated bands |
| 25 | Maltipoo | 160 | 28 | 38.6 | 8.8 | 0.4 | 6.1 | No | No |  |
| 26 | Crossbreed | 124 | 28 | 38.2 | 11.7 | 0.1 | 7.1 | No | No |  |
| 27 | Crossbreed | 200 | 20 | 38.2 | 7.1 | 1.6 | 6.8 | No | No |  |
